# Effects of Motor Versus Cognitive Task Prioritization During Dual-Task Practice on Dual-Task Performance in Young Adults

**DOI:** 10.3389/fpsyg.2020.581225

**Published:** 2020-11-12

**Authors:** Rainer Beurskens, Dennis Brueckner, Thomas Muehlbauer

**Affiliations:** ^1^Department of Health and Social Affairs, FHM Bielefeld – University of Applied Sciences, Bielefeld, Germany; ^2^Division of Movement and Training Sciences/Biomechanics of Sport, University of Duisburg-Essen, Essen, Germany

**Keywords:** postural control, stabilometer, skill acquisition, cognitive interference task, human

## Abstract

**Background**: Previous studies have reported positive effects of concurrent motor and cognitive task practice compared to motor or cognitive task practice only on dual-task performance in young adults. Knowledge about the effect of motor vs. cognitive task prioritization during practice on dual-task performance remains unclear and has not been investigated in depth so far. Thus, we examined the effects of motor task compared to cognitive task prioritization during dual-task practice on motor-cognitive performance in healthy young adults.

**Methods**: Healthy young adults were randomly assigned to dual-task (DT; i.e., concurrent motor and cognitive practice) or single-task (ST; i.e., motor or cognitive task practice only) practice groups. In DT practice, subjects were instructed to either prioritize the motor or the cognitive task. The motor task required subjects to keep a stabilometer in a horizontal position. The cognitive task involved serial three subtractions. Outcome variables were the root-mean-square error (RMSE) for the motor task and the total number of correct calculations for the cognitive task. All participants practiced for 2 consecutive days under their respective treatment condition and were tested under DT condition 24 h later (i.e., retention on day 3) without providing instructions on task prioritization.

**Results**: Irrespective of prioritization (i.e., prioritize the motor task or the cognitive task), the DT practice groups similarly improved their DT motor and cognitive task performance. The ST groups also improved motor or cognitive performance depending on their respective training contents (i.e., motor practice improved RMSE and cognitive practice improved number of correct calculations but not vice versa).

**Conclusion**: We conclude that DT compared to ST practice is well-suited to improve DT performance, irrespective of task-prioritization. DT but not ST practice resulted in an improved modulation of both domains (i.e., motor and cognitive) during DT performance. Our findings might be explained by freeing up central resources following DT practice that can be used to effectively perform the concurrent execution of motor and cognitive processing demands. However, this process is not further enhanced by the prioritized task domain.

## Introduction

Situations involving the simultaneous control of two or more tasks are the norm rather than an exception in everyday life. For example, performing a motor task (e.g., standing or walking) while concurrently being involved in a cognitive task (e.g., talking and memorizing signs) results in performance decrements in one or both of the executed tasks. Chong et al. (2010) showed that the concurrent execution of a serial subtraction task during bipedal standing had a significant detrimental effect on balance (i.e., increase in postural sway) and on cognitive (i.e., decrease in speed and accuracy) performance in healthy young adults. Further, Beauchet et al. (2005) found a significantly slower walking speed (i.e., balance performance) and a reduced number of enumerated figures (i.e., cognitive performance) under dual-task (DT) compared to single-task (ST) conditions in healthy young adults. These performance decrements are even more pronounced in older individuals and in persons with neurological disorders (i.e., stroke and cerebral palsy). Tramontano et al. (2017) found that auditory and/or visual discrimination during straight-path-walking resulted in reduced DT walking speeds. Healthy elderly subjects as well as children reduced their walking speeds during DT by 4.9–8.6% while older adults with stroke (−15.6%) and children with cerebral palsy (−15.3%) walked even slower during DT conditions.

Previous research showed that DT practice (i.e., concurrent practice of a motor and a cognitive task) is an effective training regimen to counteract decrements in DT performance (Pellecchia, 2005; Worden and Vallis, 2014). Worden and Vallis (2014) examined the impact of DT compared to ST practice on DT performance in healthy young adults. DT practice included the concurrent practice of a motor (i.e., obstacle walking) and a cognitive (i.e., auditory Stroop task) task, while ST practice consisted of practicing the motor and cognitive task separately. As a result, participants in the DT but not in the ST practice group significantly improved their motor and cognitive performance under DT test condition. It was further shown that task prioritization during DT training affected gait performance in patients with stroke (Sengar et al., 2019). Patients receiving variable instructions (i.e., prioritize either the motor or the cognitive task) learned and retained the DT faster than participants receiving instructions not prioritizing any of the tasks. These results indicate that DT practice represents an effective training regimen to improve DT performance.

The role of specific task prioritization during DT practice remains unclear and has, to our knowledge, not been investigated in intervention studies so far. This is surprising since cross-sectional studies already showed that the prioritization of one of the performed tasks during DT situations has positive effects on DT performance. For example, Schaefer et al. (2008) investigated the concurrent performance of a motor (i.e., bipedal stance on a wobble board) and a cognitive (i.e., N-back) task in young individuals. They showed that postural sway significantly decreased when participants were instructed to prioritize the motor task. In another study by Yogev-Seligmann et al. (2010), participants walked while performing a verbal fluency task. A significant increase in gait speed was detected under the instruction to focus on the motor task. Similarly, Kelly et al. (2010) showed that cognitive task performance and walking responded to task prioritization. Instructions to focus on the cognitive task resulted in shorter response latencies, while instructions to focus on walking resulted in faster gait speeds during DT walking in healthy young adults.

Reasons for the beneficial effect of task prioritization can be explained by limited cognitive capacities (i.e., “single channel model”; Pashler, 1994; Pashler and Johnston, 1998) and/or cognitive interference when two tasks share the same processing resources (i.e., “capacity sharing model”; Tombu and Jolicoeur, 2003; Wickens, 2008). Particularly, the latter theory is well-suited to explain positive effects of prioritization. The capacity sharing model argues that there is a pool of processing resources that can be distributed between different tasks. Whenever more processing resources are devoted to one task, limited processing capacity remains and tasks and performance deficits in the given tasks arise. By prioritizing one task over another, the limited resources are explicitly allocated and the prioritized task will benefit from this resource allocation. This model is also used by the “posture second” or the “posture first” (Bloem et al., 2006; Yogev-Seligmann et al., 2012) strategy claiming that the cognitive task is prioritized over the motor task, withdrawing attention from controlling posture or that the motor task is prioritized over the cognitive task. During the latter, there is no limitation of motor performance and the risk of loosing balance and falling is low.

However, although previous literature indicates that task prioritization is beneficial when being involved in DT situations, it remains open how the implementation of task prioritization during DT practice may affect DT performance in young adults. The purpose of the present study was to extend the established findings of task prioritization on DT postural control (Schaefer et al., 2008) in young adults and investigates the effect of motor vs. cognitive task prioritization during DT practice on DT performance in young adults. On the basis of the literature, our first prediction was that motor and cognitive task performance tested under DT conditions is better after DT compared to ST practice. Secondly, task prioritization during DT practice differently affects motor as well as cognitive task performance in DT conditions by improving motor task performance but not cognitive task performance after DT practice with prioritization of the motor task and vice versa.

## Materials and Methods

### Participants

**Figure 1:**
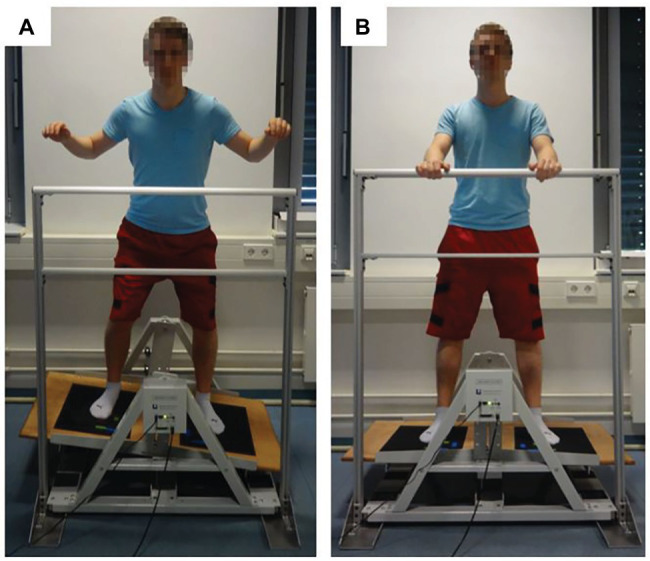
Depiction of a subject balancing **(A)** and standing **(B)** on the stabilometer (stability platform).

The present study comprised a total of 64 healthy young adults. Subjects were included if they were physically active (i.e., 2–4 h/week for at least 1 year) young adults (i.e., 20–30 years) with no known musculoskeletal, neurological, or orthopedic disorder during the last 3 months prior to the beginning of the study. Using G*Power, power analysis (*f* = 0.40, *α* = 0.05, 1-*β* = 0.70, number of groups: *n* = 4) revealed that a total sample size of *N* = 60 participants (i.e., *n* = 15 per group) would be sufficient to detect statistically significant differences. Participants had no prior experience with the experimental tasks. All subjects signed informed consent prior to the experiment. In addition, written informed consent was obtained from the individual shown in Figure 1 for the publication of any potentially identifiable images in this article. The Human Ethics Committee at the University of Duisburg-Essen, Faculty of Educational Sciences approved the study protocol.

### Tasks and Equipment

A detailed description of the methods used to assess motor and cognitive task performance has been published previously (Kiss et al., 2018). During the dynamic balance task, participants were asked to stand on a stability platform (Lafayette Instrument, Model 16,030, Lafayette, USA) consisting of a 65 cm × 107 cm wooden platform, which allowed a maximum deviation of 15° from the horizontal to either side (Figure 1). A safety rail (105 cm above the platform) mounted to the stability platform ensured that participants did not fall if they lost their balance. The motor task required participants to remain in balance (i.e., to keep the stability platform in a horizontal position) for as long as possible during each 90-s trial (Figure 1A). A timer measured time in balance at a sampling rate of 25 Hz. Time in balance was defined as time when the platform was within ±3° of horizontal position. Platform position data were exported from the analysis software PsymLab and used to calculate the root-mean-square error (RMSE) in degrees.

Cognitive performance was registered for 90 s using an arithmetic task. Participants were asked to loudly recite serial three subtractions starting from a randomly selected number between 300 and 900 that was given by the experimenter (Pellecchia, 2005). If a subject miscalculated, the false calculation was noted. When correctly continuing the serial three subtractions, only one error was noted (i.e., no consequential errors were registered). The number of correct subtractions (i.e., total number of subtractions minus the number of subtraction mistakes) was used as outcome measure. Thus, the higher the total number of correct subtractions, the better the cognitive task performance.

### Experimental Design

The experimental design of the study consisted of a 2-day practice phase followed by a delayed retention test (24 h later). Using Research Randomizer (www.randomizer.org), 64 young sex-matched adults were randomly assigned to four different practice groups: (1) ST motor group (*n* = 16; eight men, eight women; mean age: 25.3 years; SD: 2.7 years; mean body height: 174.6 cm; SD: 11.4 cm), practicing the motor task only; (2) ST cognitive group (*n* = 16; eight men, eight women; mean age: 24.6 years; SD: 4.0 years; mean body height: 176.3 cm; SD: 9.9 cm), practicing the cognitive task only; (3) DT motor prioritization group (*n* = 16; eight men, eight women; mean age: 22.9 years; SD: 2.6 years; mean body height: 178.8 cm; SD: 8.0 cm), practicing the dynamic balance and the cognitive task concurrently with prioritization of the motor task; and (4) DT cognitive prioritization group (*n* = 16; eight men, eight women; mean age: 22.0 years; SD: 2.3 years; mean body height: 176.3 cm; SD: 7.5 cm), practicing the dynamic balance and the cognitive task concurrently with prioritization of the cognitive task. At baseline, no significant differences (*p* = 0.650) regarding anthropometrics (i.e., body height) were found between the four groups.

Every trial started with the platform in horizontal position and arms grasping the safety rail (Figure 1B). Approximately 15 s before the start of a trial, the experimenter asked the participant to step on the platform without shoes. About 3 s before the start of a trial, the experimenter provided the starting number for the serial subtraction task. At the start signal, data collection started and the participant attempted to move the platform and performed the calculations.

All participants performed one familiarization trial followed by seven 90-s practice trials on each of 2 consecutive days of practice under their respective treatment conditions. A 90-s rest period was provided between trials. Knowledge of results (i.e., time in balance and/or total number of correct calculations) was provided after each trial. Concerning participants in the two ST practice groups, the instructions were as follows: *“Please keep the platform as horizontal as possible and try to remain in a stable position in the given trial”* (ST motor) and *“Please perform as many serial three subtractions as possible in the given trial”* (ST cognitive). Regarding participants in the two DT practice groups, the instructions on task prioritization were given prior to the first, third, fifth, and seventh trial and were as follows: *“Please keep the platform as horizontal as possible and try to remain in a stable position and perform as many serial three subtractions as possible in the given trial and thereby prioritize the motor task”* (DT motor prioritization) and *“Please keep the platform as horizontal as possible and try to remain in a stable position and perform as many serial three subtractions as possible in the given trial and thereby prioritize the cognitive task”* (DT cognitive prioritization). On day 3 (i.e., 24 h later), all participants were tested under DT condition (retention) using one 90-s trial; yet neither instructions on task prioritization nor knowledge of results were provided.

### Statistical Analysis

During acquisition on days 1 and 2, the RMSE was analyzed in a 3 (group: ST motor, DT motor prioritization, DT cognitive prioritization) × 2 (day: day 1–2) × 7 (trial: trial 1–7) analysis of variance (ANOVA) with repeated measures on days and trials. Additionally, the total number of correct calculations during acquisition was analyzed in a 3 (group: ST cognitive, DT cognitive prioritization, DT motor prioritization) × 2 (day: day 1–2) × 7 (trial: trial 1–7) ANOVA with repeated measures on days and trials. During testing on day 3 (retention), the RMSE and the total number of correct calculations obtained under DT condition were analyzed using a one-way ANOVA. In case of statistically significant group differences, Bonferroni corrected *post hoc* Student’s *t*-tests were conducted. In addition, Cohen’s *d* was calculated to determine whether a statistical difference was practically meaningful as small (0 ≤ *d* ≤ 0.49), medium (0.50 ≤ *d* ≤ 0.79), or large (*d* ≥ 0.80). Normal distribution was examined using the Kolmogorov-Smirnov test (*p* ≥ 0.20). All analyses were performed using the Statistical Package for Social Sciences (SPSS) version 24.0 and the level of significance was set at *p* < 0.05.

## Results

**Figure 2:**
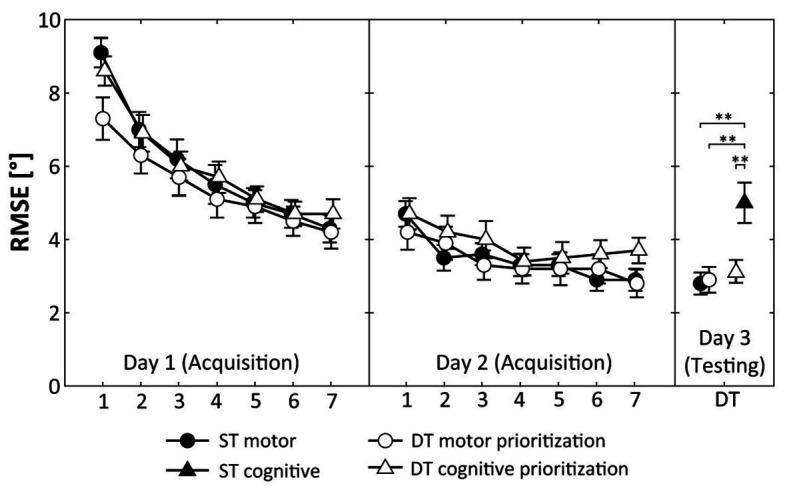
Root mean square error (RMSE) during acquisition (days 1 and 2) and DT retention testing (day 3). Values represent means and standard errors by group. ST, single-task; DT, dual-task; ^**^ indicate *p* < 0.01 for *post hoc* comparisons.

**Figure 3:**
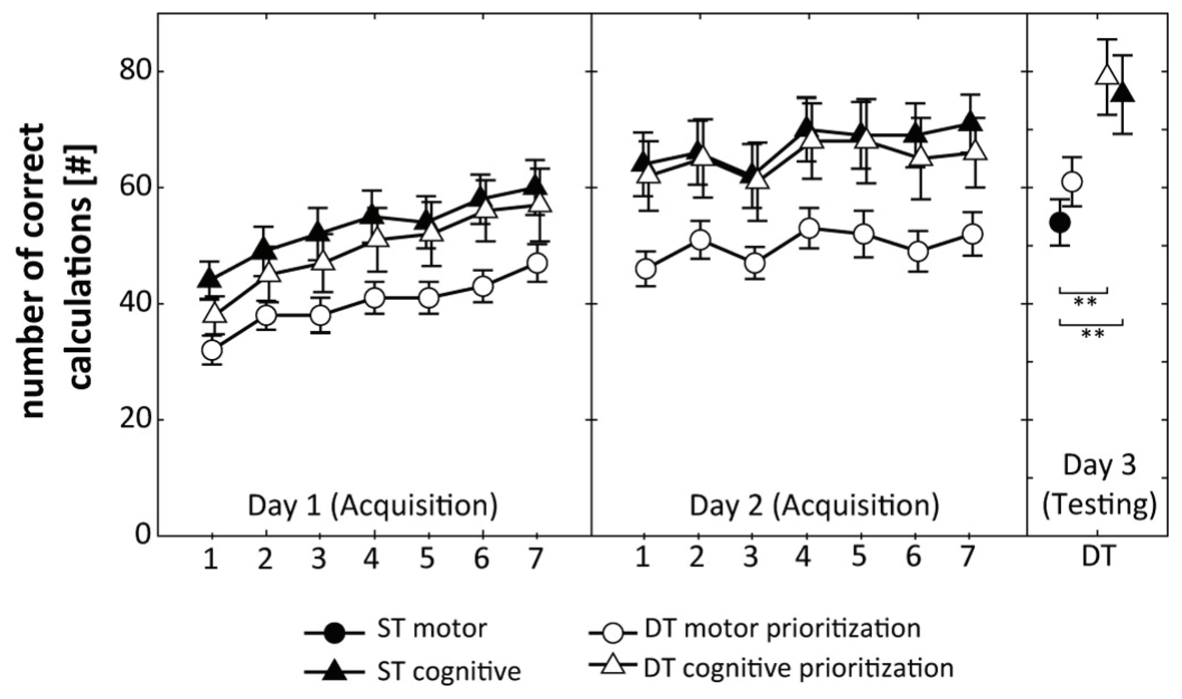
Total number of correct calculations during acquisition (days 1 and 2) and DT retention testing (day 3). Values represent means and standard errors by group. ST, single-task; DT, dual-task; ^**^ indicate *p* < 0.01 for *post hoc* comparisons.

All participants received the practice condition as initially allocated and no participant dropped out. Results are displayed in Figure 2 (representing motor performance, i.e., RMSE) and Figure 3 (representing cognitive performance, i.e., total number of correct calculations).

### Acquisition (days 1 and 2)

Figure 2 shows that participants of the ST motor group, the DT motor prioritization group, and the DT cognitive prioritization group decreased their RMSE across the 2 days of practice. ANOVA revealed a significant Day × Trial interaction (*F*_(6, 45)_ = 40.150, *p* < 0.001, *d* = 1.89), indicating relatively greater improvements on day 1 than on day 2. The Group × Day × Trial interaction (*F*_(12, 270)_ = 1.589, *p* = 0.094, *d* = 0.53) did not reach the level of significance.

Figure 3 displays that participants of the ST cognitive group, the DT cognitive prioritization group, and the DT motor prioritization group increased their total number of correct calculations over the 2 practice days. The respective ANOVA detected a significant Day × Trial interaction (*F*_(6, 45)_ = 13.859, *p* < 0.001, *d* = 1.11), indicating relatively greater improvements on day 1 than on day 2. The Group × Day × Trial interaction (*F*_(12, 270)_ = 0.612, *p* = 0.832, *d* = 0.33) did not reach the level of significance.

### Retention Testing (Day 3)

Regarding motor performance, one-way ANOVA showed significant differences between the groups (*F*_(3, 60)_ = 7.114, *p* < 0.001, *d* = 1.13). *Post hoc* comparisons indicated significantly smaller RMSE values in DT condition for the ST motor group (*p* = 0.002, *d* = 1.22), the DT motor prioritization group (*p* = 0.003, *d* = 1.16), and the DT cognitive prioritization group (*p* = 0.005, *d* = 1.06) compared to the ST cognitive group (Figure 2). No further significant differences were detected.

Regarding cognitive performance, one-way ANOVA revealed significant differences between the groups (*F*_(3, 60)_ = 4.772, *p* = 0.005, *d* = 1.00). *Post hoc* comparisons indicated significantly larger total numbers of correct calculations under the DT condition for the ST cognitive (*p* = 0.008, *d* = 1.00) and the DT cognitive prioritization group (*p* = 0.003, *d* = 1.16) compared to the ST motor group (Figure 3). No other comparison revealed statistical significance.

## Discussion

In the present study, we examined the effects of motor vs. cognitive task prioritization during DT practice on DT performance in healthy young adults. Main findings regarding DT testing (retention) on day 3 can be summarized as follows: (i) task prioritization during DT practice did not affect motor and cognitive task performance differentially; (ii) irrespective of task prioritization, DT practice resulted in improvements in both task domains (i.e., motor and cognitive); (iii) improvements during ST practice were limited to the trained task (i.e., ST cognitive training improved number of correct calculations in DT condition and ST motor training improved RMSE in DT condition). During practice phase (i.e., days 1 and 2), both DT groups and the respective ST group improved performance in motor control and/or in cognitive task execution. That is, ST motor and both DT groups (motor prioritization as well as cognitive prioritization group) decreased RMSE during standing on the stability platform. Further, ST cognitive and both DT groups (motor prioritization and cognitive prioritization group) increased the number of correct calculations.

### Effects of Dual Compared to Single-Task Practice

For the two DT practice groups, motor task performance was not different compared to the ST motor group but was significantly better compared to the ST cognitive group when tested under DT condition. Further, both DT practice groups’ did not differ in the cognitive task but performed better than the ST motor group in a DT test situation. Thus, we replicated the results of our previous study (Kiss et al., 2018), which showed that DT but not ST practice resulted in motor and cognitive task improvements when tested under DT condition. Based on these findings, it is suggested that DT practice effectively modulates both domains (i.e., motor and cognitive), whereas ST practice is effective to modulate the trained domain (i.e., motor or cognitive) only. In other words, DT but not ST practice seems to be a suitable training regimen to positively affect motor and cognitive processing capacities during DT situations. Indeed, it has been shown that DT practice induces structural (Takeuchi et al., 2014) and functional (Garner and Dux, 2015) changes in the human brain, indicating a more effective modulation of central processing mechanisms. Takeuchi et al. (2014) highlight increased gray matter volume in several brain regions (i.e., prefrontal cortical regions, left posterior parietal cortex, and the left temporal and lateral occipital areas) following 4 weeks of multitask training using auditory stimuli. Garner and Dux (2015) identified specific regions in frontoparietal as well as subcortical areas of the human brain that showed increased activity in response to multitasking demands following training. These finding indicate that DT practice is suitable to elicit DT specific adaptations in the human brain enabling it to effectively adapt to DT demands. These adaptations might also free up attentional resources during DT conditions that can then be used to process concurrent demands (i.e., motor vs. cognitive task). Thomson et al. (2020) found that switching attention between multiple tasks affected gait stability in young adults. Authors suggest that switching attention resulted in a posture second prioritization strategy during DT walking. The need to perform two tasks simultaneously increases deficits in gait stability due to the switching of attentional resources between both tasks (Siu et al., 2008, 2009; Hawkes et al., 2012).

### Effects of Task Prioritization During Dual-Task Practice

Irrespective of task prioritization, DT practice did not affect motor and cognitive tasks performance differentially when tested under DT condition. This is contrary to our second hypothesis. Given that there is no study available that scrutinized the effects of motor compared to cognitive task prioritization during DT practice on DT performance in young adults, our results have to be compared with results originating from studies using similar training regimens (i.e., DT balance training with fixed vs. variable task prioritization; Silsupadol et al., 2009a,b). For example, Silsupadol et al. (2009a) investigated effects of ST balance training compared to DT balance training with either fixed or variable task prioritization on DT performance in older adults. After 4 weeks of practice, DT balance training with variable task prioritization was more effective in improving both motor (i.e., gait behavior) and cognitive (i.e., rate of response) performance under DT conditions than either DT balance training with fixed task prioritization or ST balance training. Further, Silsupadol et al. (2009b) examined DT performance following 4 weeks of ST balance training and DT balance training with fixed vs. variable task prioritization in elderly persons. They found larger motor (i.e., gait speed) and cognitive (i.e., successful trial number) task improvements in a DT test situation for participants who received DT balance training with variable task prioritization compared to those with fixed task prioritization and compared to the ST balance training group.

What are likely explanations for our observation that task prioritization during DT practice did not significantly enhance the prioritized domain in a DT test situation? It could be argued that processing demands needed for the respective task prioritization were rather low, challenging none of the two domains explicitly. Thus, both domains (i.e., motor and cognitive) improved during DT practice, irrespective of task prioritization. In other words, maintaining dynamic postural control on a moveable platform while simultaneously performing serial three subtractions can be quick and automatically processed in young adults. Since automated tasks excel through good task performance, prioritization of one or the other task did not lead to further performance improvements. This argument is supported by the capacity sharing model stated by Tombu and Jolicoeur (2003) and Wickens (2008). The low demands of our cognitive and/or motor task lead to low demands on central capacities in young adults. The pool of processing resources in young adults that can be distributed between different tasks was not exceeded by serial three subtractions and processing resources could still be devoted to both tasks simultaneously. Further, one might argue that studies by Silsupadol et al. (2006, 2009a,b) used a DT balance training already applying different balance (e.g., standing on foam and firm surfaces and narrow and backward walking) as well as cognitive demands (e.g., naming objects, remembering numbers and spelling words) and thus incorporated varying stimuli as well as high attentional demands. In contrast to these findings, the presented study applied the same balance (i.e., balancing on a stabilometer) and cognitive task (i.e., serial three subtractions). As a consequence, stimuli as well as attentional demands did not change or vary in our study, leading to low attentional resources needed for the task prioritization.

Different approaches have been used to explain the effects of task prioritization. First, the “posture second” strategy introduced by Bloem et al. (2006) claims that the cognitive task is prioritized over the motor task. This strategy withdraws attention from controlling posture, thereby increasing the risk of loosing balance and falling. Another approach states that priority of the motor is given over the cognitive task (i.e., “posture first” strategy; Bloem et al., 2006; Yogev-Seligmann et al., 2012). Thus, there is no explicit limitation of motor performance during DT practice and the risk of loosing balance and falling is low. Our results are not in line with the first or with the second approach. Task prioritization did not differently favor task performance in the prioritized domain during DT balance performance in young adults. One might again argue that central capacity in healthy young adults is not stressed enough by challenging demands during DT situations. Thus, their cognitive capacity in terms of attentional resources is able to adequately handle both, the motor demand of the stabilometer task as well as the cognitive demand of serial three subtractions. More difficult and attention-demanding tasks during DT conditions might overstrain central capacity in young adults. For example, in older adults it has been shown that particularly visual demands cause greater reductions in DT motor performance compared to other (e.g., verbal or auditive) tasks (Bock, 2008; Beurskens and Bock, 2012). Shumway-Cook et al. proposed that the allocation of attention during the performance of concurrent tasks is complex, depending on many factors including the nature of the cognitive task, the postural task, the goal of the subject, and the instructions, implying that task prioritization is flexible and depends on a variety of individual, task, and environmental factors (Shumway-Cook et al., 1997; Kelly et al., 2013). This finding might be used to explain our results, indicating that the motor and/or cognitive task used itself did not affect prioritization. Consequently, other aspects, such as individual preconditions (e.g., physique, cognitive status and motivation) might affect DT performance following prioritized practice. However, this needs further clarification in terms of DT practice and task prioritization. Further, other studies showed immediate effects of task prioritization during DT conditions (Schaefer et al., 2008; Kelly et al., 2010; Yogev-Seligmann et al., 2010). Our results did not show differences in motor and/or cognitive performance following specific test instructions, indicating that prioritization of one over another task is beneficial during task execution. This however did not lead to a specific learning effect during the retention/transfer phase of the experiment.

There are a few limitations in this study that have to be addressed. First, the allocation of subjects to our sample groups needs clarification. We used a pseudo-randomized trial, meaning that participants were randomly allocated to one of the four groups. However, the main population that was used to recruit subjects consisted of sports science students. Thus, we only included fit and physically active young students that may not be representative for the general population of healthy young adults and might have performed better in the motor-cognitive task based on their higher level of physique compared to non-active young adults. Additionally, the balance task represents a fairly specific setting that is predominantly used by physical therapists but is not commonly used in the field of sports and everyday life, which limits the transferability of our findings to these situations. Furthermore, the presented results only apply to healthy young adults in the investigated age-group. Future studies are advised to also compare different populations. For example, balance performance is known to decrease with advancing age and further deteriorates in persons with clinical impairments (i.e., neurological deficits).

## Conclusion

This is the first study that examined the effects of motor compared to cognitive task prioritization during DT practice on DT performance in healthy young adults. Results suggested that DT practice leads to an improved modulation of both domains (i.e., motor and cognitive), whereas ST practice elicits an enhanced modulation of the trained domain (i.e., motor or cognitive) only. Further, task prioritization during DT practice does not enhance the prioritized domain under a DT test condition differentially indicating that transfer or retention is not achieved by 2-day-practice. Nevertheless, results of the study provide a framework for interpreting changes in DT performance. Improving our understanding of DT performance has potential clinical implications during the assessment of DT deficits associated with aging and neurologic disorders and the development of suitable treatment options to mitigate these deficits.

## Data Availability Statement

The raw data supporting the conclusions of this article will be made available by the authors, without undue reservation.

## Ethics Statement

The studies involving human participants were reviewed and approved by Human Ethics Committee at the University of Duisburg-Essen, Faculty of Educational Sciences. The patients/participants provided their written informed consent to participate in this study. Written informed consent was obtained from the individual for the publication of any potentially identifiable images in this article.

## Author Contributions

TM and RB developed the research design and were the primary authors of the manuscript. DB collected the data and gave edits throughout the creation of the manuscript. All authors contributed to the article and approved the submitted version.

### Conflict of Interest

The authors declare that the research was conducted in the absence of any commercial or financial relationships that could be construed as a potential conflict of interest.

## References

[ref1] Beauchet O. Dubost V. Herrmann F. R. Kressig R. W. (2005). Stride-to-stride variability while backward counting among healthy young adults. J. Neuroeng. Rehabil. 2:26. 10.1186/1743-0003-2-26, PMID: 16095533 PMC1190208

[ref2] Beurskens R. Bock O. (2012). Age-related deficits of dual-task walking: a review. Neural Plast. 2012:131608. 10.1155/2012/131608, PMID: 22848845 PMC3403123

[ref3] Bloem B. Grimbergen Y. van Dijk J. Munneke M. (2006). The posture second strategy: a review of wrong priorities in Parkinson’s disease. J. Neurol. Sci. 248, 196–204. 10.1016/j.jns.2006.05.010, PMID: 16806270

[ref4] Bock O. (2008). Dual-task costs while walking increase in old age for some, but not for other tasks: an experimental study of healthy young and elderly persons. J. Neuroeng. Rehabil. 13, 27–36. 10.1186/1743-0003-5-27, PMID: 19014544 PMC2596160

[ref5] Chong R. K. Mills B. Dailey L. Lane E. Smith S. Lee K. H. (2010). Specific interference between a cognitive task and sensory organization for stance balance control in healthy young adults: visuospatial effects. Neuropsychologia 48, 2709–2718. 10.1016/j.neuropsychologia.2010.05.018, PMID: 20478320

[ref6] Garner K. G. Dux P. E. (2015). Training conquers multitasking costs by dividing task representations in the frontoparietal-subcortical system. Proc. Natl. Acad. Sci. U. S. A. 112, 14372–14377. 10.1073/pnas.1511423112, PMID: 26460014 PMC4655506

[ref7] Hawkes T. D. Siu K. C. Silsupadol P. Woollacott M. H. (2012). Why does older adults’ balance become less stable when walking and performing a secondary task? Examination of attentional switching abilities. Gait Posture 35, 159–163. 10.1016/j.gaitpost.2011.09.001, PMID: 21964051 PMC3251721

[ref8] Kelly V. E. Eusterbrock A. J. Shumway-Cook A. (2013). Factors influencing dynamic prioritization during dual-task walking in healthy young adults. Gait Posture 37, 131–134. 10.1016/j.gaitpost.2012.05.031, PMID: 22940543 PMC3513485

[ref9] Kelly V. E. Janke A. A. Shumway-Cook A. (2010). Effects of instructed focus and task difficulty on concurrent walking and cognitive task performance in healthy young adults. Exp. Brain Res. 207, 65–73. 10.1007/s00221-010-2429-6, PMID: 20931180 PMC3058115

[ref10] Kiss R. Brueckner D. Muehlbauer T. (2018). Effects of single compared to dual task practice on learning a dynamic balance task in young adults. Front. Psychol. 9:311. 10.3389/fpsyg.2018.00311 29593614 PMC5857582

[ref11] Pashler H. (1994). Dual-task interference in simple tasks: data and theory. Psychol. Bull. 116, 220–244. 10.1037/0033-2909.116.2.220, PMID: 7972591

[ref12] Pashler H. Johnston J. C. (1998). “Attentional limitations in dual-task performance” in Attention. ed. Pashler H. (Erlbaum, UK: Taylor and Francis/Psychology Press), 155–189.

[ref13] Pellecchia G. L. (2005). Dual-task training reduces impact of cognitive task on postural sway. J. Mot. Behav. 37, 239–246. 10.3200/JMBR.37.3.239-246, PMID: 15883121

[ref14] Schaefer S. Krampe R. T. Lindenberger U. Baltes P. B. (2008). Age differences between children and young adults in the dynamics of dual-task prioritization: body (balance) versus mind (memory). Dev. Psychol. 44, 747–757. 10.1037/0012-1649.44.3.747, PMID: 18473641

[ref15] Sengar S. Raghav D. Verma M. Alghadir A. H. Iqbal A. (2019). Efficacy of dual-task training with two different priorities instructional sets on gait parameters in patients with chronic stroke. Neuropsychiatr. Dis. Treat. 15, 2959–2969. 10.2147/NDT.S197632 31695387 PMC6805250

[ref16] Shumway-Cook A. Woollacott M. H. Kerns K. A. Baldwin M. (1997). The effects of two types of cognitive tasks on postural stability in older adults with and without a history of falls. J. Gerontol. A Biol. Sci. Med. Sci. 52, M232–M240. 10.1093/gerona/52a.4.m232, PMID: 9224435

[ref17] Silsupadol P. Lugade V. Shumway-Cook A. van Donkelaar P. Chou L. S. Mayr U.. (2009a). Training-related changes in dual-task walking performance of elderly persons with balance impairment: a double-blind, randomized controlled trial. Gait Posture 29, 634–639. 10.1016/j.gaitpost.2009.01.006, PMID: 19201610 PMC2707497

[ref18] Silsupadol P. Shumway-Cook A. Lugade V. van Donkelaar P. Chou L. S. Mayr U.. (2009b). Effects of single-task versus dual-task training on balance performance in older adults: a double-blind, randomized controlled trial. Arch. Phys. Med. Rehabil. 90, 381–387. 10.1016/j.apmr.2008.09.559, PMID: 19254600 PMC2768031

[ref19] Silsupadol P. Siu K. C. Shumway-Cook A. Woollacott M. H. (2006). Training of balance under single-and dual-task conditions in older adults with balance impairment. Phys. Ther. 86, 269–281. PMID: 16445340

[ref20] Siu K. C. Catena R. D. Chou L. S. van Donkelaar P. Woollacott M. H. (2008). Effects of a secondary task on obstacle avoidance in healthy young adults. Exp. Brain Res. 184, 115–120. 10.1007/s00221-007-1087-9, PMID: 17717655 PMC2556305

[ref21] Siu K. C. Chou L. S. Mayr U. van Donkelaar P. Woollacott M. H. (2009). Attentional mechanisms contributing to balance constraints during gait: the effects of balance impairments. Brain Res. 1248, 59–67. 10.1016/j.brainres.2008.10.078, PMID: 19028462 PMC3133742

[ref22] Takeuchi H. Taki Y. Nouchi R. Hashizume H. Sekiguchi A. Kotozaki Y.. (2014). Effects of multitasking-training on gray matter structure and resting state neural mechanisms. Hum. Brain Mapp. 35, 3646–3660. 10.1002/hbm.22427, PMID: 24343872 PMC4216411

[ref23] Thomson D. Gupta A. Liston M. (2020). Does attention switching between multiple tasks affect gait stability and task performance differently between younger and older adults? Exp. Brain Res. 10.1007/s00221-020-05938-0 [Epub ahead of print] 33025032

[ref24] Tombu M. Jolicoeur P. (2003). A central capacity sharing model of dual-task performance. J. Exp. Psychol. Hum. Percept. Perform. 29, 3–18. 10.1037//0096-1523.29.1.3, PMID: 12669744

[ref25] Tramontano M. Morone G. Curcio A. Temperoni G. Medici A. Morelli D.. (2017). Maintaining gait stability during dual walking task: effects of age and neurological disorders. Eur. J. Phys. Rehabil. Med. 53, 7–13. 10.23736/S1973-9087.16.04203-9, PMID: 27575014

[ref26] Wickens C. D. (2008). Multiple resources and mental workload. Hum. Factors 50, 449–455. 10.1518/001872008X288394, PMID: 18689052

[ref27] Worden T. A. Vallis L. A. (2014). Concurrent performance of a cognitive and dynamic obstacle avoidance task: influence of dual-task training. J. Mot. Behav. 46, 357–368. 10.1080/00222895.2014.914887, PMID: 24914575

[ref28] Yogev-Seligmann G. Hausdorff J. M. Giladi N. (2012). Do we always prioritize balance when walking? Towards an integrated model of task prioritization. Mov. Disord. 27, 765–770. 10.1002/mds.24963, PMID: 22419512

[ref29] Yogev-Seligmann G. Rotem-Galili Y. Mirelman A. Dickstein R. Giladi N. Hausdorff J. M. (2010). How does explicit prioritization alter walking during dual-task performance? Effects of age and sex on gait speed and variability. Phys. Ther. 90, 177–186. 10.2522/ptj.20090043, PMID: 20023000 PMC2816029

